# OsSNDP3 Functions for the Polar Tip Growth in Rice Pollen Together with OsSNDP2, a Paralog of OsSNDP3

**DOI:** 10.1186/s12284-022-00586-0

**Published:** 2022-07-20

**Authors:** Sunok Moon, Yu-Jin Kim, Ha Eun Park, Junhyup Kim, Yun Shil Gho, Woo-Jong Hong, Eui-Jung Kim, Su Kyoung Lee, Byung-Chang Suh, Gynheung An, Ki-Hong Jung

**Affiliations:** 1 Department of Genetic Engineering and Crop Biotech Institute, Kyung Hee University, Yongin, 17104 Korea; 2 Department of Life Science and Environmental Biochemistry, and Life and Industry Convergence Research Institute, Pusan National University, Miryang-si, 50463 Korea; 3 Department of Brain Sciences, DGIST, Daegu, 42988 Korea

## Abstract

Understanding pollen tube growth is critical for crop yield maintenance. The pollen tube provides a path for sperm cells for fertilization with egg cells. Cells must be subdivided into functionally and structurally distinct compartments for polar tip growth, and phosphoinositides are thought to be one of the facilitators for polarization during pollen tube growth. *OsSNDP3* encodes Sec14-nodulin domain-containing protein and localizes in the nucleus and the microdomains of the plasma membrane in tobacco leaf epidermis cells. OsSNDP3 is thought to bind with phosphatidylinositol 4,5-bisphosphate based on the data including the information of basic amino acids in the C-terminal and colocalization with 2X Pleckstrin homology domain of Phospholipase C *delta*-1. OsSNDP3 interacts with a protein that contains a class I nodulin domain. We discovered that OsSNDP3 plays a significant role in pollen tube germination using CRISPR/Cas9 systems, whereas another pollen-preferential Sec14-nodulin domain-containing protein, OsSNDP2, additively functions with OsSNDP3 during pollen tube germination. Gene Ontology analysis using downregulated genes in *ossndp3* indicated that the expression of genes involved in the phosphatidylinositol metabolic process and tip growth was significantly altered in *ossndp3*. OsSNDP3 aids pollen polar tip growth by binding with phosphatidylinositol 4,5-bisphosphate. We can better understand the roles of phosphoinositides during pollen tube growth by studying the functions of OsSNDP3 and OsSNDP2. And downregulated genes in *ossndp3* might be useful targets for future research on polar tip growth.

**Supplementary Information:**

The online version contains supplementary material available at 10.1186/s12284-022-00586-0.

## Background

Pollen germination is a very critical step for fertilization. For successful fertilization in rice, pollens have to start germination within a few minutes after shedding (Moon and Jung 2020; Pacini and Dolferus 2019). Rice mature pollen grain is made up of a large vegetative cell and two sperm cells enclosed with vegetative cell (Jiang et al. 2015). Vegetative cells control pollen germination, and pollen tubes provide a pathway for sperm cells to reach the embryo sac. Rice pollen tube elongates at a rate of up to 5 mm/h by polarized tip growth (Chen et al. 2008; Qin and Yang 2011). Several regulatory mechanisms governing polar tip growth have been identified (Qin and Yang 2011). Polarization requires the cell to be divided into functionally and structurally distinct compartments (Krahn and Wodarz 2012). Proteins and lipids are, in fact, asymmetrically distributed at the plasma membrane (Tejos et al. 2014).

Phosphoinositides (PIs) are distinctly distributed membrane phospholipids and make up less than 1% of the cellular lipid cohort (Krahn and Wodarz 2012; Shewan et al. 2011). In eukaryotic cells, chemically distinct seven PIs are produced by phosphorylation of the myo-inositol head group of phosphatidylinositol (PtdIns) at positions 3, 4, and 5 (Shewan et al. 2011). PtdIns synthase is in charge of the biosynthesis of PtdIns in the endoplasmic reticulum (Kim et al. 2011). PIs are only found on the cytosolic face of membranes and are differentially enriched in membrane compartments (Grabon et al. 2019; Krahn and Wodarz 2012). For example, in animals, phosphatidylinositol 4,5-bisphosphate [PtdIns(4,5)P2] and phosphatidylinositol 3,4,5-trisphosphate [PtdIns(3,4,5)P3] are mutually exclusively localized at the plasma membrane (Krahn and Wodarz 2012; Gassama-Diagne et al. 2006). Phosphatidylinositol 4-phosphate (PtdIns4P) and PtdIns(4,5)P2 are specifically enriched in plant root cell apical and basal plasma membranes (Tejos et al. 2014). They participate in polar localization of cargo like as PIN-FORMED, auxin transporters (Tejos et al. 2014). Furthermore, PtdIns4P and PtdIns(4,5)P2 were found to be specifically localized at the tip-growing plasma membrane of root hairs and pollen tubes (Braun et al. 1999; Thole et al. 2008; Ischebeck et al. 2008). PtdIns(4,5)P2 is synthesized from PtdIns4P by PtdIns4P 5-kinases (Hempel et al. 2017). Mitogen-activated protein kinases (MAPK) and Rac/ROP GTPase are involved in the synthesis of PtdIns(4,5)P2, via phosphorylating or recruiting of PtdIns4P 5-kinases in the apical plasma membrane of pollen tubes, respectively (Hempel et al. 2017; Krishnamoorthy et al. 2014). PtdIns(4,5)P2 is hydrolyzed by Phospholipase C (PLC) into inositol 1,4,5-trisphosphate [Ins(1,4,5)P3] and diacylglycerol (DAG), removing PtdIns(4,5)P2 as a polarized recruitment signal from the plasma membrane (Stenzel et al., 2020). DAG can be more converted to phosphatidic acid (PA) through DAG kinase (Munnik 2001). PA is also produced by Phospholipase D (PLD) via hydrolysis of phosphatidylcholine (Munnik 2001). PtdIns(4,5)P2, Ins(1,4,5)P3 and PA modulate tip-focused calcium gradient, membrane secretion, and cytoskeleton organization, thus playing a key role in the maintenance of polarity (Monteiro et al. 2005).

Local accumulation of PIs in plasma membrane induces the recruitment of specific protein-containing lipid-binding domains (Alessi et al. 1997; Mao and Yin 2007). Pleckstrin homology (PH), phagocyte oxidase homology (PX), FYVE and SEC14 domains mediate interactions between proteins with PIs (Krahn and Wodarz 2012). The SEC14-like phosphatidylinositol transfer protein (Sec14-like PITP) superfamily was discovered by screening for secretory mutants in yeast (Novick et al. 1980; Vincent et al. 2005). There are 32 and 27 genes encoding Sec14-like PITP in *Arabidopsis* and rice, respectively (Huang et al. 2016a). Whereas Sec14 homologues are mainly expressed as the single-domain Sec14 proteins in yeast, multi-domain Sec14 proteins containing either GOLD domain or nodulin domain were predominantly found in *Arabidopsis* and rice genome (Huang et al. 2016a). In vitro PtdIns or PtdCho transfer activities are detected from single-domain Sec14 proteins (Soybean Sec14 Homologs 2 and AtPITP11) and multi-domain Sec14 proteins (AtSFH1, AtSFH2, AtSFH4, AtSFH5 and AtSFH9) (Huang et al. 2016b; Kearns et al. 1998). Apart from these lipid transfer activities, Sec14-like PITPs are involved in PIs synthesis by executing an interfacial presentation of PtdIns to PtdIns 4-OH kinases (Huang et al. 2016a).

*Arabidopsis* AtSfh1is the best studied Sec14-like PITP and encodes the multi-domain Sec14 protein with nodulin domain (Ghosh et al. 2015). Sec14-nodulin domain-containing proteins are divided into three classes by their extreme C-terminal sequences (Ghosh et al. 2015). Class I subfamily proteins of Sec14-nodulin domain-containing proteins have an uninterrupted stretch of > 7 basic amino acids in the C-terminal with vicinal aromatic residues, but basic residues are contiguous in C-terminal of class II and class III (Ghosh et al. 2015). The only class I subfamily proteins are targeted to the plasma yeast membrane in a PtdIns(4,5)P2-dependent manner among fourteen Sec14-nodulin domain-containing proteins from *Arabidopsis* (Ghosh et al. 2015). AtSfh1, one of the class I nodulin domain-containing protein, binds to PtdIns(4,5)P2 via the nodulin domain and the basic amino is in charge of binding with PtdIns(4,5)P2 (Ghosh et al. 2015). AtSfh1 maintains tip-directed PtdIns(4,5)P2 gradients for polarized tip growth and membrane trafficking in developing root hairs (Ghosh et al. 2015; Vincent et al. 2005). OsSNDP1 is identified as orthologue of AtSfh1 and it is involved in root hair elongation from rice (Huang et al. 2013).

In this study, we discovered that *OsSNDP3* is required for pollen tube elongation. It is thought that OsSNDP3 binds to PtdIns(4,5)P2 and regulates polar tip growth. We also provide RNA-seq analysis of downregulated genes in the *ossndp3* mutant, which may be useful targets for understanding polar tip growth.

## Results

### Pollen-Preferential Sec14-Nodulin Domain-Containing Protein3, OsSNDP3, is Essential for Gene Transfer Through the Pollen

**Fig. 1:**
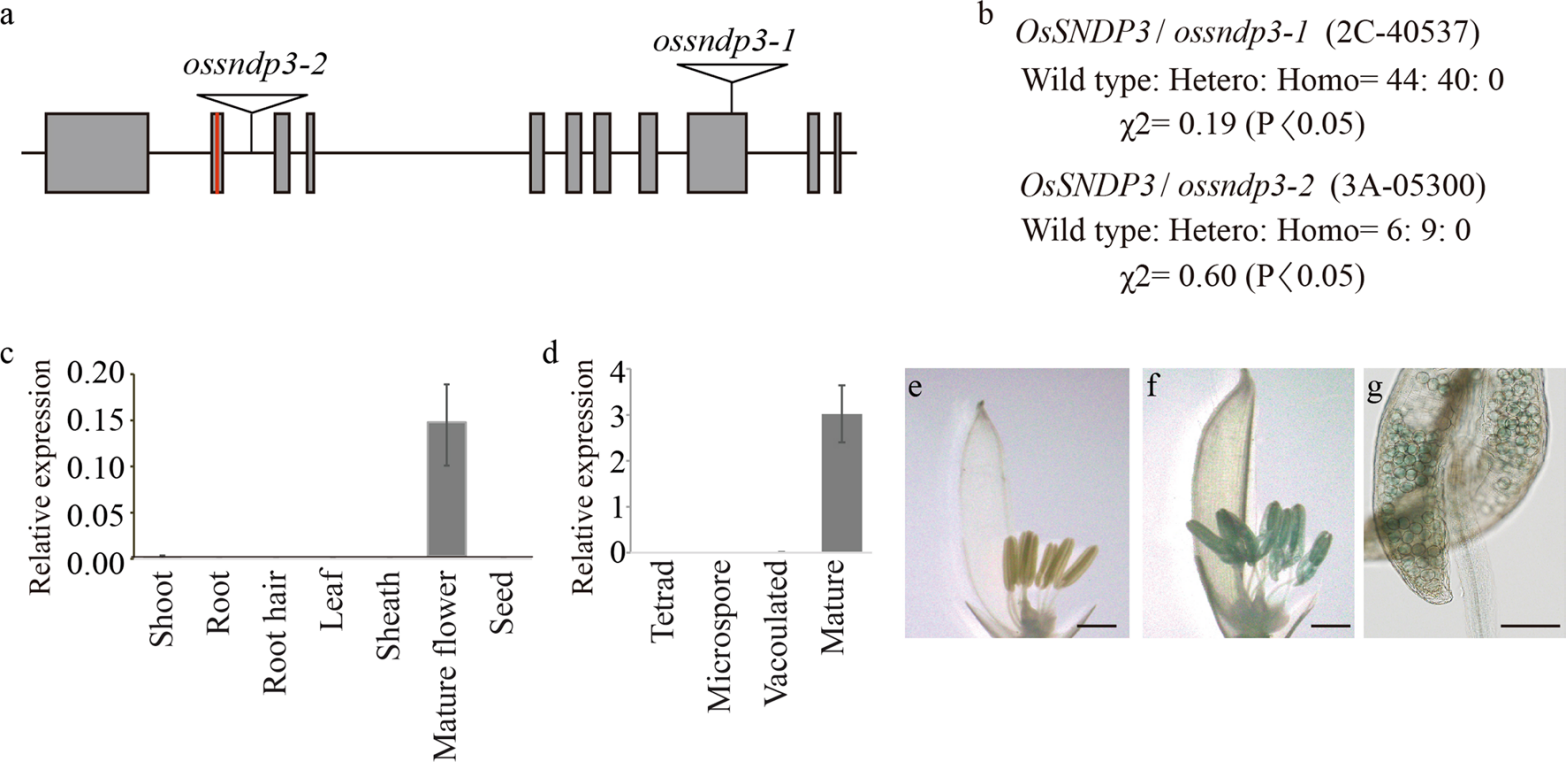
The expression pattern of *OsSNDP3*. **a** Schematic representation of *OsSNDP3* including T-DNA insertion and target region for gene-editing. Grey boxes indicate exon; lines, intron; reverse triangles, T-DNA; red box, target region of CRISPR/Cas9. **b**. Segregation analysis of *OsSNDP3*/*ossndp3-1* and *OsSNDP3*/*ossndp3-2*, and results of Chi-square test (χ2)*.* RT-qPCR analysis of *OsSNDP3* in various tissues (**c**) and different stages of anther development (**d**). Histochemical GUS analysis of transgenic plants harboring p*OsSNDP3*::GUS vector. Pictures were taken from the vacuolated (**e**), mature (**f**), and anthesis stage (**g**) flowers. Bars = 1 mm (**e** and **f**) and 500 µm (**g**)

We previously conducted a genome-wide analysis of late pollen-preferred genes and tried to identify key genes involved in gene transfer via genotyping T-DNA insertion lines for 627 candidate genes (Moon et al. 2018). As a result, mutants showing distortions in segregation ratios that were close to 1:1:0 (wild type: heterozygote: mutant homozygote) were selected for further studies. Among the mutants, T-DNA insertion in a mutant was identified in the Sec14-nodulin domain-containing protein3 (*OsSNDP3*, *LOC_Os02g04030*) (Huang et al. 2016a) (Fig. 1a). We named this mutant as *ossndp3-1*. We found another allele (*ossndp3-2*) from the T-DNA insertion mutants population. This line also exhibited a 1:1:0 segregation (Fig. 1b). We performed reciprocal crosses between heterozygotes (*OsSNDP3*/*ossndp3-1*) and wild type (WT) to see if OsSNDP3 functioned in male gametophyte or female gametophyte. When *OsSNDP3*/*ossndp3-1* was used as female, WT and heterozygous descendants were obtained at a similar frequency (Table 1). But when *OsSNDP3*/*ossndp3-1* heterozygote plant was used as pollen donner, just WT progenies were obtained (Table 1). This data showed that *ossndp3-1* successfully transfer the gene through the female gametophyte but not the male.

**Table 1:** Genetic transmission analysis of *OsSNDP3*/*ossndp3-1* by reciprocal crossing. The segregation ratio was analyzed using F1 progenies of represented crosses. The transmission efficiency (TE) represents the percentage of transmitted ossndp3 mutant alleles through male or female gametes.

Genotype (female × male)	Wild type	Hetero	TE
*OsSNDP3*/*ossndp3-1* × WT	10	13	130.0
WT X *OsSNDP3*/*ossndp3-1*	40	0	0.0

To check the expression pattern of *OsSNDP3*, we used reverse transcription-quantitative PCR (RT-qPCR). Whereas transcript of *OsSNDP3* was seldom detected in vegetative organs and seeds, it is abundantly presented in mature flowers (Fig. 1c). *OsSNDP3* expression increased dramatically during the subsequent anther development stages (Fig. 1d). Following that, we created transgenic plants containing GUS reporter genes under the control of the *OsSNDP3* promoter. Whereas no GUS staining was found in pollens at the vacuolated stage, it was detected in pollens at the mature stage (Fig. 1e–g).

### OsSNDP3 Colocalized with 2X PH Domain of Phospholipase C *Delta*-1

**Fig. 2:**
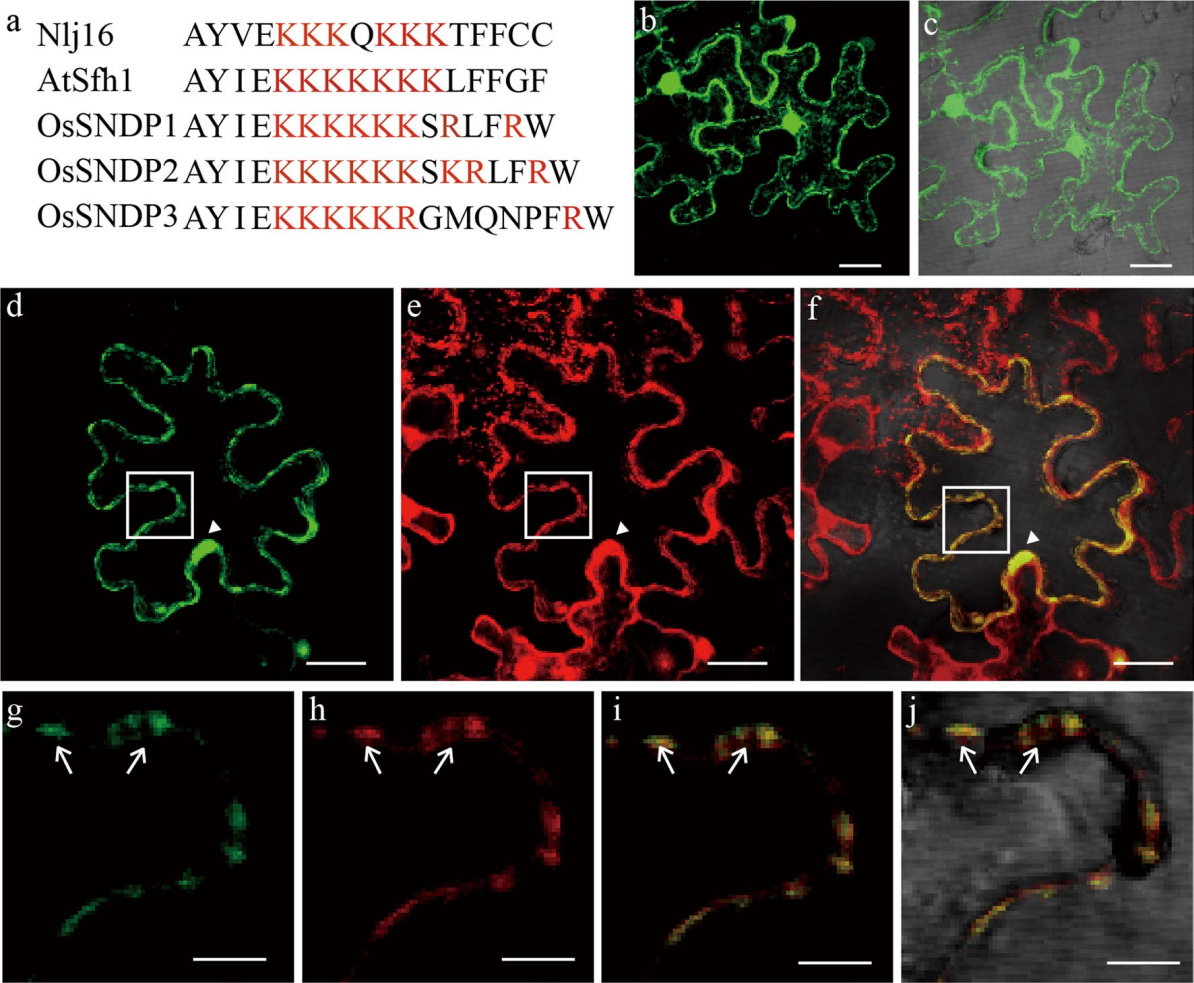
Subcellular localization of OsSNDP3. **a** Alignments of the C-termini of Class I nodulin domains protein. Basic amino acids are represented as red. Cells expressing 35S::GFP as control (**b** and **c**). Plasma membrane and nucleus (triangle) localization of GFP–OsSNDP3 (**d**), RFP–2X PH domain of PLCδ1 (**e**), and merged image (**f**). Highly magnified image (**g**–**j**). Arrow indicated microdomains of the plasma membrane. GFP and RFP signals appeared as green and red. Bars = 20 µm (**b**–**f**) and 5 µm (**g**–**j**)

Class I nodulin domain-containing proteins interact with PtdIns(4,5)P2 through the basic amino acid within a nodulin domain and localized at the plasma membrane of yeast in a PtdIns(4,5)P2-dependent manner (Ghosh et al. 2015). In the C-terminal of OsSNDP3, there is uninterrupted stretch of six basic amino acids (Fig. 2a). Uninterrupted stretch of basic amino acids is found in three the Sec14-nodulin domain-containing protein from rice (Fig. 2a). Because only Class I nodulin domain-containing proteins from *Arabidopsis* are targeted to the plasma membrane, we analyzed localization of OsSNDP3. To investigate OsSNDP3 subcellular localization, we created a GFP-OsSNDP3 fusion construct under the control of the 35S promoter. As a marker, we created a fusion construct of RFP and the 2X PH domain of Phospholipase C delta-1 (PLCδ1) from *Rattus norvegicus*, which binds with PtdIns(4,5)P2 in the plant (Simon et al. 2014). 35S::GFP is used as control. *Agrobacterium* infiltrated tobacco leaf epidermis cells, causing them to express GFP, GFP-OsSNDP3 and RFP-2X PH. Whereas green fluorescence from 35S::GFP detected throughout the cell (Fig. 2b–c), fluorescence from both GFP-OsSNDP3 and RFP-2X PH were appeared in the nucleus and the plasma membrane (Fig. 2d–f). Most signals from GFP-OsSNDP3 were overlapped with those from RFP-2X PH (Fig. 2f). Signals were detected at the plasma membranes microdomains, indicating that they were not distributed evenly within the plasma membrane (Fig. 2g–j).

### OsSNDP3 is Essential for Pollen Tube Growth

**Fig. 3:**
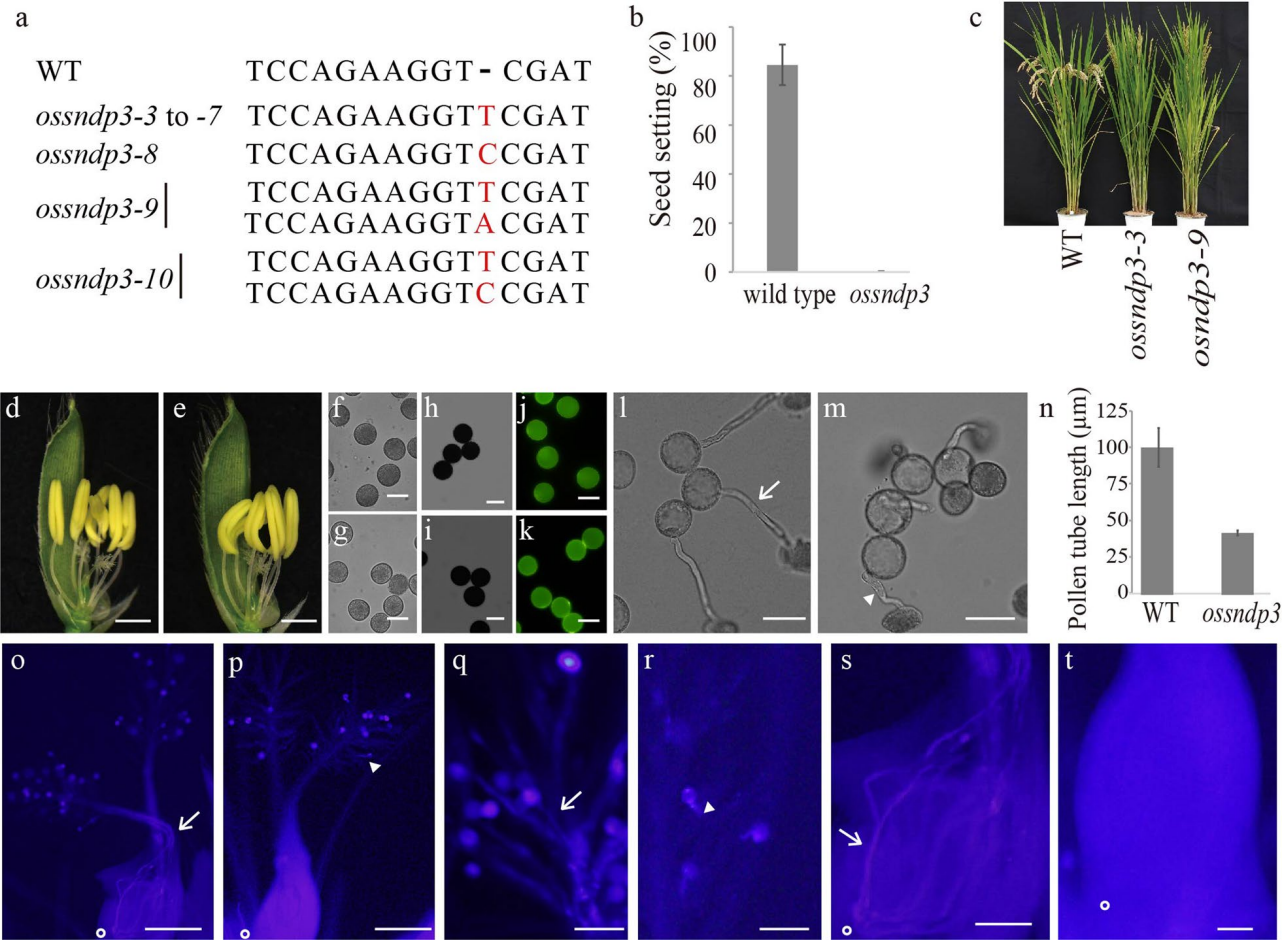
The mutant phenotype of *OsSNDP3*. **a** Sequencing analysis of the target sites of CRISPR/Cas9 on the genomic regions of *OsSNDP3*. Red characters indicate mutated sequences. **b** Fertilities of nine panicles from wild type and *ossndp3*. Each three panicles used from *ossndp3-3*, *ossndp3-4* and *ossndp3-*5. **c** Picture of wild type, *ossndp3-3* and *ossndp3-9* at ripening stage. Flower structure (**d** and **e**), pollen in PBS (**f** and **g**) and pollen stained by Lugol’s iodine (**h** and **i**), and Auramine O (**j** and **k**) from the WT (**d**, **f**, **h,** and **j**) and *ossndp3-3* (**e**, **g**, **i,** and **k**)*.* In vitro pollen germination of wide type (**l**) and *ossndp3-3* (**m**). Pictures were taken 30 min after germination on solid germination media. Arrow represented long pollen tube from wild type and triangle indicated short pollen tube from *ossndp3-3*. **n** pollen tube length from wild type and *ossndp3-3*. Values are means (± SE) of pollen tube lengths (n > 25 pollen tubes). In vivo pollen germination stained by aniline blue (**o**–**t**). The pollens on stigma germinate through the style and enter the micropyle (open circle) in wild type (**o**, **q,** and **s**). The short pollen tube (triangle) stayed in the stigma of *ossndp3-3* (**p**, **r,** and **t**). Bars = 1 mm (**d** and **e**), 50 µm (**f**–**m**), 500 µm (**o** and **p**), and 100 µm (**q**–**t**)

We were unable to obtain a homozygotic mutant due to a defect of *ossndp3* in gene transfer through the male gamete. We used the CRISPR-Cas9 system to create homozygotic mutants of *OsSNDP3*. We obtained six monoallelic (from *ossndp3-3* to *ossndp3-8*) and two biallelic null mutants (*ossndp3-9* and *ossndp3-10*) through sequencing analysis (Fig. 3a). These null mutants were sterile (Fig. 3b and c). Except for sterility, any difference was not detected between WT and *ossndp3* (Fig. 3b and c).

In mature flowers, anthers were filled with pollens in both WT and *ossndp3* (Fig. 3d and e). We collected pollens from mature anther and stained pollens using Lugol’s iodine (to detect starch) or auramine O (to detect exine) (Fig. 3f–k). There was no difference between WT pollen and mutant pollen, indicating that *ossndp3* pollen matured normally. As a result, we anticipate that the *ossndp3* mutant will have a defect in pollens after maturation. We then examined pollen germination in vitro. The germination efficiency of WT and *ossndp3* is approximately 75% (75.6% for wild type and 74.4% for *ossndp3*). Whereas WT pollen produces an elongated tube, *ossndp3* mutant only has a short tube (Fig. 3l and m). For quantitative analysis, we measured the lengths of pollen tubes from WT and *ossndp3*. When compared to the WT, the length of the pollen tube decreases by 41% in *ossndp3* (Fig. 3n). Aniline blue staining was used to examine pollen germination in WT and *ossndp3* plants in vivo (Fig. 3o–t). In contrast, pollen tubes of WT reach the embryo sac to fertilize with the egg cell in WT (Fig. 3o, q, and s), pollen tube of *ossndp3* stayed in stigma tissue (Fig. 3p, r, and t). These data indicated that *ossndp3* is essential for pollen tube growth in vivo.

### OsSNDP3 Interacts with Class I Nodulin Domain-Containing Protein

**Fig. 4:**
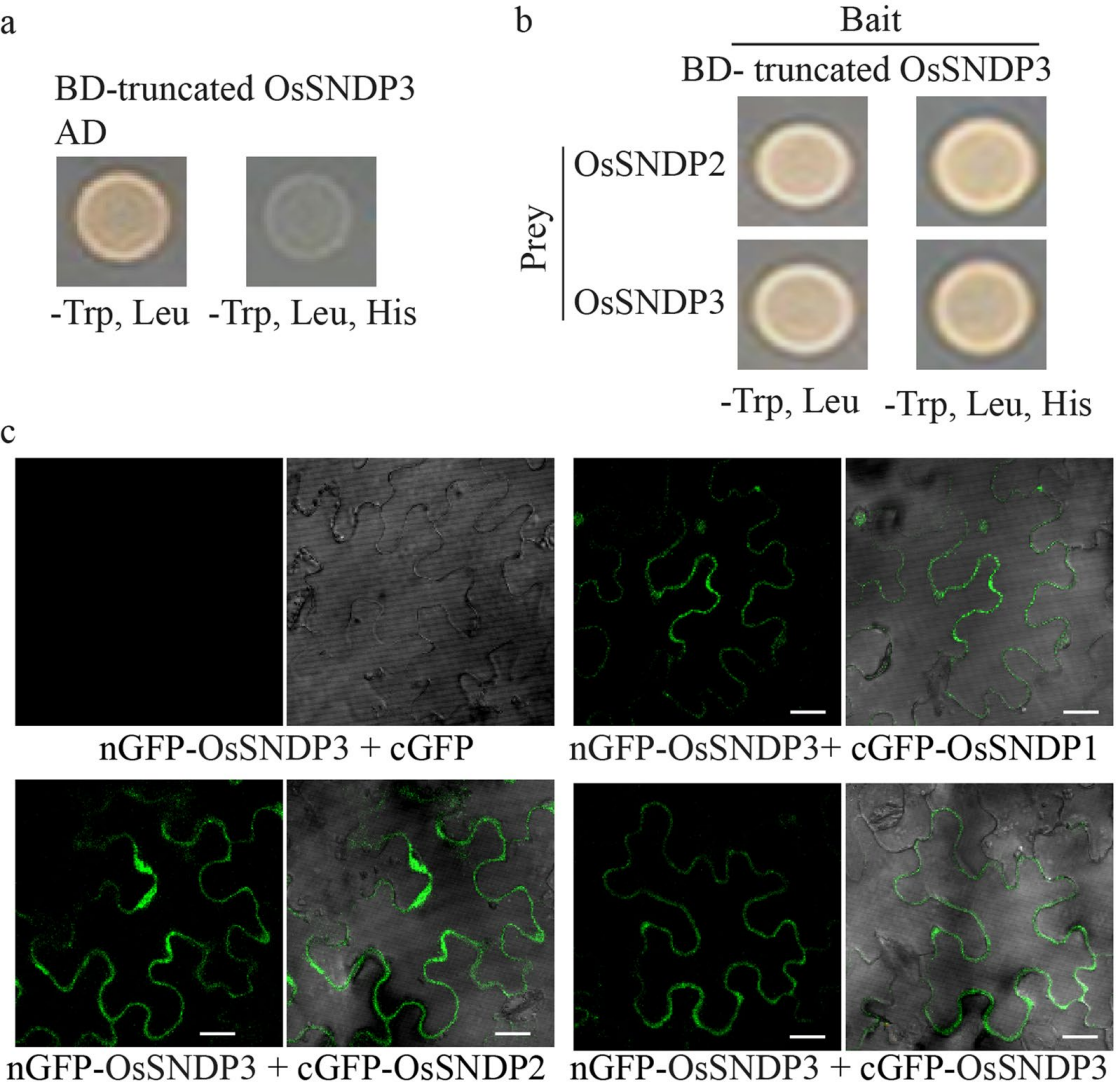
Interaction between OsSNDP3 and class I nodulin domain-containing proteins. **a** Yeast self-activation assay of truncated OsSNDP3. **b** Yeast two-hybrid analysis to confirm the interaction between OsSNDP3 and identify prey proteins. **c** Interactions between OsSNDP3 and class I nodulin domain-containing proteins by BiFC. Fluorescent signals were detected from a cell expressing nGFP-OsSNDP3/cGFP, nGFP-OsSNDP3/cGFP-OsSNDP1, nGFP-OsSNDP3/cGFP-OsSNDP2, and nGFP-OsSNDP3/ cGFP-OsSNDP3. Bars = 10 µm

To get a clue about the function of OsSNDP3 during pollen germination, we performed yeast two hybrids (Y2H) screening. Yeast cells expressing both the activation domain (AD) and the GAL4 binding domain (BD) fused with full-length OsSNDP3 or truncated OsSNDP3 were unable to grow on synthetic minimal (SM) media lacking Trp, Leu, and His (Fig. 4a). We did not obtain any valuable interactors when we used full-length SNDP3 as a bait. We obtained five positive clones corresponding to genes, OsSNDP2 and OsSNDP3, using truncated OsSNDP3 (containing nodulin domain) as a bait. Truncated OsSNDP3 and identified prey proteins were again introduced into yeast once more and dropped on SM media lacking Trp, Leu, and His (Fig. 4b). Yeast coexpressing BD-Truncated OsSNDP3/AD-OsSNDP2 or BD-Truncated OsSNDP3/AD-OsSNDP3 grew well, indicating OsSNDP3 interacts with OsSNDP2 or OsSNDP3 (Fig. 4b).

To confirm the interactions between OsSNDP3 and class I nodulin domain-containing proteins, we performed bimolecular fluorescence complementation (BiFC) assay using tobacco cells. Three class I nodulin domain-containing proteins were fused with either the N- or the C-terminal fragment of a split GFP molecule for this assay. As negative control, we used nGFP-OsSNDP3 and cGFP. Whereas any signal was not detected from negative control experiment, fluorescent signals were detected from a cell expressing nGFP-OsSNDP3 and cGFP-OsSNDP1 (Fig. 4c). Combinations of nGFP-OsSNDP3/cGFP-OsSNDP2 and nGFP-OsSNDP3/cGFP-OsSNDP3 produced similar fluorescent signals, indicating that class I nodulin domain proteins interact with each other (Fig. 4c).

### OsSNDP3 and OsSNDP2 Function Additively During Pollen Tube Germination

**Fig. 5:**
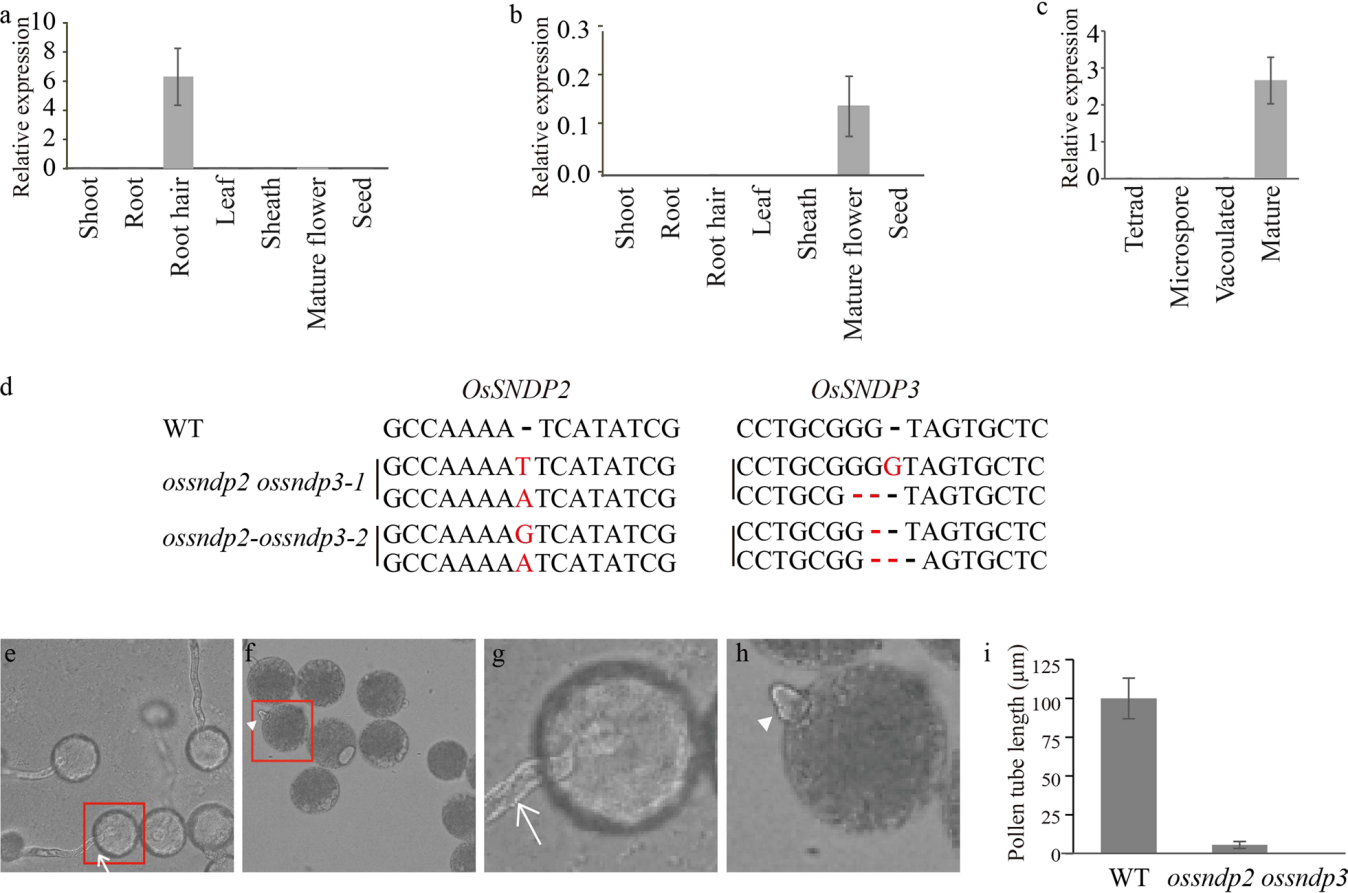
Double mutant phenotype of *ossndp2 ossndp3*. The expression pattern of *OsSNDP1* (**a**) and *OsSNDP2* (**b** and **c**). **d** Sequencing analysis of the target region of CRISPR/Cas9 on *OsSNDP2* and *OsSNDP3* from double mutants. Red characters indicate inserted sequences by the gene-editing system and red dashes indicate deleted sequences. In vitro pollen germination from wide type (**e** and **g**) and *ossndp2 ossndp3* (**f** and **h**). Pictures were taken 30 min after germination on the solid germination media. Arrow indicated long pollen tube from wild type and triangle marked protrusion of pollen tube. Magnified images of the red box were shown in **g** and **h**. Representative images were taken from *ossndp2 ossndp3-1.* **i** pollen tube length from wild type and *ossndp2 ossndp3*. Values are means (± SE) of pollen tube lengths (n > 20 pollen tubes)

We found that the three class I nodulin domain-containing proteins in rice, OsSNDP1, 2, and 3, interacted with each other. To check their contribution during pollen germination, we checked the expression of OsSNDP1 and OsSNDP2 using RT-qPCR. Transcripts of *OsSNDP1* are highly detected in root hairs and *OsSNDP2* was abundantly expressed in mature flowers (Fig. 5a and b). During the anther developmental stage, *OsSNDP2* expression was similar to that of *OsSNDP3* (Fig. 5c). OsSNDP1 is thought to be involved in the polarized tip growth of root hair (Huang et al. 2013). To learn more about *OsSNDP2*, we created homozygotic mutants with the CRISPR-Cas9 system, but seed setting rate from the homozygotic mutants of *OsSNDP2* was similar to that from wild type (Additional file 1: Fig. S1). From this data, we guessed that *ossndp2* is not defective in gene transfer through the male or female gamete.

We generated a double mutant of *OsSNDP2* and *OsSNDP3* using the CRISPR-Cas9 system. There were just two double homozygotic mutants for *OsSNDP2* and *OsSNDP3* and we named these mutants as *ossndp2 ossndp3-1* and *ossndp2 ossndp3-2* (Fig. 5d). There was no difference between WT pollen and mutant pollen (Additional file 1: Figure S2). In vitro, pollen germination tests revealed that *ossndp2 ossndp3* pollens had protruded pollen tubes but not elongated pollen tubes (Fig. 5e–h). The double mutants pollen tube growth is more defective than that of *ossndp3*. When compared to the WT, the length of the pollen tube reaches 6% in *ossndp2 ossndp3* (Fig. 5i)*.* Although *OsSNDP2* does not have a critical role during pollen germination, it works in conjunction with *OsSNDP3* during pollen tube germination.

### RNA-Seq Analysis of Ossndp3 Anthers versus WT Anthers Suggests OsSNDP3 Involved in Phosphoinositide Signaling and Tip Growth

**Fig. 6:**
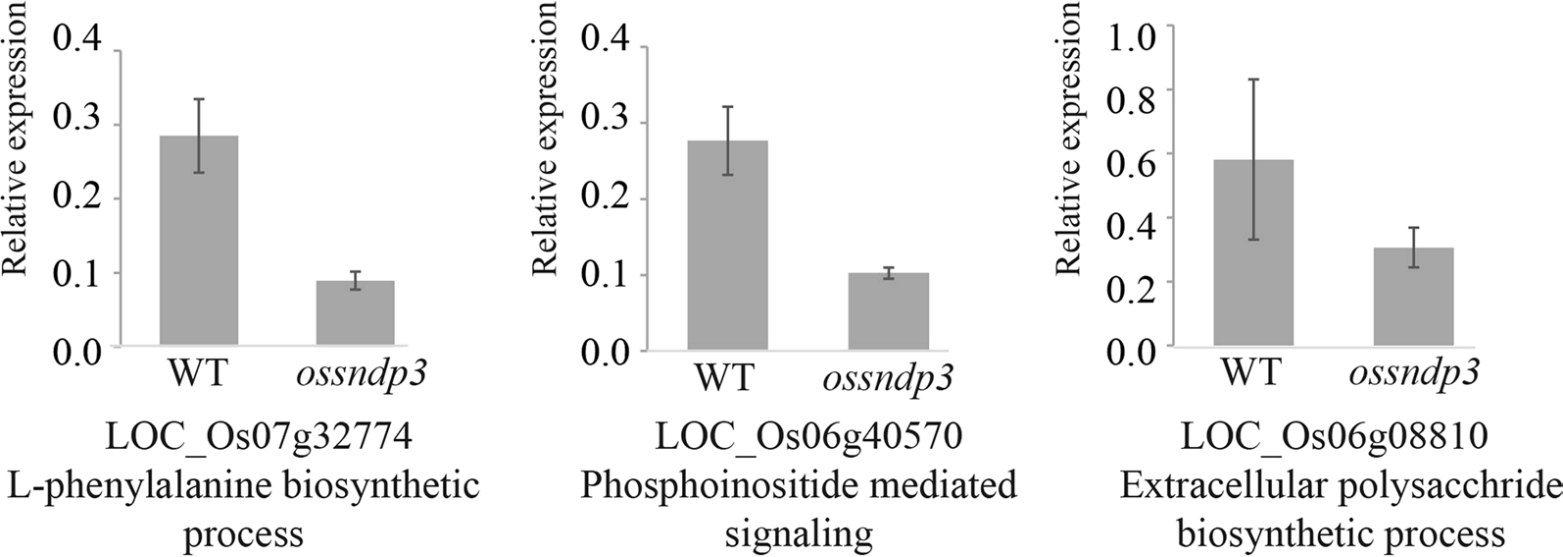
Real-time RT-qPCR analysis of 3 genes downregulated in *ossndp3.* The relative Expression level of *LOC_Os07g32774*, *LOC_Os06g40570,* and *LOC_Os06g08810* in mature anthers of wild type and *ossndp3*

*OsSNDP3* was highly expressed at mature anther and the defect was firstly observed at the pollen germination stage. We compared the transcriptomes of *ossndp3* anthers with WT anthers to identify potential candidate genes influenced by *OsSNDP3*. Anthers at the mature stage were used for RNA-seq analysis because mature pollens contain transcripts for pollen germination. Based on the following three criteria: Fragments Per Kilobase of transcripts per Million mapped reads (FPKM) in WT mature anther ≥ 100; log_2_ fold change ≤  − 1; and p-values ≤ 0.05, we selected 661 downregulated genes in *ossndp3* compared with that in WT (Additional file 2: Table S1). Gene Ontology (GO) enrichment tool installed in Rice Oligonucleotide Array Database (ROAD; http://ricephylogenomics-khu.org/ROAD/analysis/go_enrichment.shtml; Cao et al., 2012) was used for GO terms in the biological process category (Additional file 2: Table S2). Among significantly enriched GO terms with fold change ≥ 2, p-values ≤ 0.05, GO terms involved in phosphoinositide-mediated signaling (29.5) and phosphatidylinositol metabolic process (8.2) were over-represented in the downregulated genes by *OsSNDP3* mutation (Table 2). Furthermore, *ossndp3* was linked to GO terms related to tip growth, such as the extracellular polysaccharide biosynthetic process and vesicle docking involved in exocytosis (Table 2). The GO term related to the L-phenylalanine biosynthetic process (13.4) was enriched in *ossndp3* (Table 2)*.* To validate RNA-seq data, we used RT-qPCR to examine and confirm the expression patterns of three genes in mature anthers from WT and *ossndp3* (Fig. 6).

**Table 2:** The analysis of significantly enriched Gene Ontology terms for downregulated genes in *ossndp3* anthers compared to wild type anther during maturation stage

GO category	No. of GO repeats^a^	No. of GO repeats in queried genes^b^	No. of expected GO repeats^c^	Fold-enrichment value^d^
Phosphoinositide-mediated signaling	5	2	0.1	29.5
Guanosine tetraphosphate metabolic process	8	2	0.1	18.4
L-phenylalanine biosynthetic process	11	2	0.1	13.4
Extracellular polysaccharide biosynthetic process	23	4	0.3	12.8
Vesicle docking involved in exocytosis	12	2	0.2	12.3
Cell wall modification	46	7	0.6	11.2
Cation transport	81	10	1.1	9.1
Phosphatidylinositol metabolic process	18	2	0.2	8.2
Trehalose biosynthetic process	28	3	0.4	7.9
Steroid biosynthetic process	69	6	0.9	6.4
Actin cytoskeleton organization	25	2	0.3	5.9
Ciliary or flagellar motility	63	5	0.9	5.8
One-carbon metabolic process	28	2	0.4	5.3
Cellular cell wall organization	132	8	1.8	4.5
DNA topological change	94	5	1.3	3.9
Dellular metabolic process	133	7	1.8	3.9
Carbohydrate metabolic process	600	25	8.1	3.1
Vesicle-mediated transport	112	4	1.5	2.6
Potassium ion transport	203	6	2.8	2.2

The total number of GO terms in the rice genome is 39,571 and queried the number of GO terms in downregulated genes in *ossndp3* is 537

^a^Several selected GO Slim terms annotated in the rice genome

^b^Observed the number of selected GO Slim terms in queried downregulated genes in *ossndp3*

^c^Expected number of selected GO Slim terms in queried downregulated genes in *ossndp3*

^d^Relative ratio of an observed number to expected number for a selected GO Slim term

## Discussion

### OsSNDP3 Functions During Pollen Tube Growth

Three class I nodulin domain-containing proteins are preferentially expressed in the tip-growing tissues of rice (Figs. 1 and 5). Two of them in particular, *OsSNDP2* and *OsSNDP3* are highly expressed in mature pollen and interact with one another. We were identified OsSNDP2 and OsSNDP3 as the interactors of OsSNDP3 from yeast two hybrid analysis using truncated OsSNDP3 (Fig. 4b). And BiFC experiment revealed that full length OsSNDP3 interacts with class I nodulin domain-containing proteins (Fig. 4c). We guessed that the failure of yeast experiment using full length OsSNDP3 came from plasma membrane localization of OsSNDP3. Because all Class I subfamily proteins of Sec14-nodulin domain-containing proteins from *Arabidopsis* are targeted into plasma membrane in yeast, full length protein of these family is not suitable for yeast two hybrid analysis. (Ghosh et al. 2015). For successful yeast two hybrid, bait-prey complex has to transport into nucleus. Because Sec14-nodulin domain-containing proteins are classified by their extreme C-terminal sequences and Class I subfamily is considered as novel polarity regulator, we additionally used C-terminal region of SNDP3 (508–633) for yeast two hybrid analysis and got two positive interactors (Fig. 4b). AtSfh1 functions as tetramer and oligomerization is important for enhancements of PtdIns(4,5)P2 binding and plasma membrane localization of AtSfh1 (Ghosh et al. 2015). Thus, interaction between OsSNDP3 and Class I subfamily may be important to execute their function as novel polarity regulator. Despite similarity of expression pattern between OsSNDP2 and OsSNDP3 and the mutual interaction, *OsSNDP3* has a more exclusive function during pollen tube growth than *OsSNDP2*. It is critical to identify factors that cause functional differences between OsSNDP2 and OsSNDP3 to better understand the role of class I nodulin domain-containing proteins.

PtdIns(4,5)P2 is asymmetrically distributed at the pollen tube plasma membrane (Tejos et al. 2014). Because *OsSNDP3* is a pollen-preferentially expressed gene and is thought to function during pollen tube growth, it may be more useful to understand OsSNDP3 localization during pollen tube elongation. Although we looked at the localization of OsSNDP3 in tobacco leaf epidermal cells, the signals completely matched with 2X PH of PLCδ1. Because 2X PH of PLCδ1 is indicator of PtdIns(4,5)P2, OsSNDP3 is colocalized with PtdIns(4,5)P2 in tobacco leaf epidermal cells. As depicted at *atsfh1* losing tip-directed PtdIns(4,5)P2 gradients in root hair, distribution of PtdIns(4,5)P2 might be altered in *ossndp3* mutant pollen during pollen tube growth (Ghosh et al. 2015). A result of the loss of tip-directed PtdIns(4,5)P2 gradients causes mislocalization of cargo for tip growth.

### RNA-Seq Analysis of *Ossndp3* Provides the Candidate Genes to Study on Pollen Tube Growth

Besides PtdIns(4,5)P2 directly regulates a wide range of cellular functions, it can be used as a substrate to produce second messengers (Stenzel et al., 2020). PtdIns(4,5)P2 also modulates tip-focused calcium gradient. OsSNDP3 might be involved in tip-directed PtdIns(4,5)P2 gradients. These features of PtdIns(4,5)P2 enable OsSNDP3-mediated gene expression regulation. To identify potential downstream candidate genes influenced by *OsSNDP3*, we carried out RNA-seq analysis using *ossndp3* and wild type anthers. Significantly enriched GO terms included phosphoinositide-mediated signaling (29.5) and tip growth. And also, the GO term related to L-phenylalanine biosynthetic process was significantly enriched in *ossndp3*. Prephenate dehydratases might be responsible for the enrichment of the L-phenylalanine biosynthetic process. Three of the nine prephenate dehydratases in rice are preferentially expressed in pollen. Although two prephenate dehydratases were selected as downregulated genes under criteria to select differentially expressed genes, three pollen-preferential prephenate dehydratases are downregulated in *ossndp3* (Additional file 2: Table S3). Phenylalanine is the common precursor of volatile benzenoids, flavonoid pigments, and other secondary metabolites with diverse functions (Sheehan et al. 2012). Although the function of flavonoids during pollen tube germination in rice has been established, more research is needed on the functions of prephenate dehydratases during pollen development (Wang et al., 2020).

Pollen-preferential ATPases are another enriched GO term related to cation transport. Pollen tubes are made up of highly polarized tip-growing cells and exhibit cytosolic pH gradients. *Arabidopsis *plasma membrane proton ATPases mutants had lower extracellular proton and cytosolic pH, resulting defect in pollen germination (Hoffmann et al. 2020). In rice, three pollen-preferential ATPases were downregulated by the mutation of *OsSNDP3* (Additional file 2: Table S4). Because OsSNDP3 is a critical component for pollen tube growth, downregulated genes revealed by RNA-seq would be useful for future research.

#### Conclusion

Polar tip growth of pollen tube is essential for successful fertilization of plant. PtdIns(4,5)P2 functions as a polarized recruitment signal from the plasma membrane. In this study, we discovered OsSNDP3 regulating polar tip growth via binding with PtdIns(4,5)P2. Through the transcriptome analysis, we also provide downregulated genes in *ossndp3* which might be useful targets for understanding polar tip growth in rice.

## Methods

### Identification of Mutants

We selected T-DNA insertional mutants having mutations within late pollen-preferred genes (Jeon et al. 2000; An et al.^2003^). DNA was extracted and PCR was performed for genotyping at seven days after germination on a half-strength Murashige and Skoog medium. The primer sequences used for genotyping of T-DNA insertional mutants for *OsSNDP3* were listed in Additional file 2:Table S5.

### Production of Homozygous Mutant via CRISPR/Cas9 System

To create a single guide (sg) RNA-Cas vectors, one set of oligomers targeting the second exon of *OsSNDP3* was synthesized. The synthesized 24 bp oligos were ligated with a Cas9 expression backbone vector pRGEB32 (Addgene plasmid ID: 63,142). Ligation products were transformed into *E*. *coli*. And then, the construct was transformed into *A*. *tumefaciens* LBA4404. The Agrobacterium-mediated stable transformation was used to create transgenic plants. Genomic DNA was extracted from transgenic plants, and PCRs were carried out with gene-specific primers. To check for mutations, PCR products were purified and sequenced.

### Cytochemical Analysis of Pollens

For staining of exine, 0.001% auramine O in 17% sucrose was used and observed under the FITC channel of the Olympus BX61 microscope. Starch was stained with Lugol’s iodine. Fresh pollen grains were collected directly from open flowers and placed on a solid pollen germination medium (PGM) to study pollen germination in vitro. The solidified PGM contained 20% (w/v) sucrose, 10% polyethylene glycol (PEG) 4000, 3 mM calcium nitrate, 40 mg L^−1^ boric acid, 10 mg L^−1^ vitamin B1, and 1% agarose. Pollen germinated at 28 °C in the dark for 30 min.

### Localization of SNDP3 in Tobacco Leaves

OsSNDP3 and 2X PH domain of PLCδ1–RFP were amplified and fused with modified pH7FWG2 and pH7RWG2. Additional file 2: Table S5 contains a list of the Primer sequences used in this study for vector constructions. A linker between PH domains was designed based on the sequences of pRS414-7 × 2-PHO5-GFP-hPLC delta PH domain dimer (Addgene plasmid ID: 58,837). GFP-SNDP3 and RFP-2X PH domain of PLCδ1 constructs were introduced into *A*. *tumefaciens* GV3101 and used for *Nicotiana benthamiana* infiltration experiments. The leaves were examined 72 h after infiltration with a Carl Zeiss LSM 510 META confocal scanning laser microscope.

### Generation of SNDP3 Promoter GUS Transgenic Plants and Histochemical GUS Assay

The promoter region of *OsSNDP3* was PCR amplified and inserted into the binary vector P3. The construct was transformed into *A*. *tumefaciens* LBA4404. Transgenic plants were obtained via Agrobacterium-mediated transformation (Jeon et al. 2000). Flowers from transgenic plants were opened using forceps and were incubated overnight at 37 °C in GUS staining solution (100 mM sodium phosphate, pH 7, 5 mM potassium ferricyanide, 5 mM potassium ferrocyanide, 0.5% Triton X-100, 10 mM EDTA, pH 8, 0.1% 5-bromo-4-chloro-3-indolyl-β-d-GlcA/cyclohexylammonium salt, 2% dimethyl sulfoxide, and 5% methanol). Chlorophyll was removed using 70% ethanol, and the stained tissues were examined using a light microscope (Olympus).

### Yeast Two-Hybrid Analysis

Full-length cDNAs and truncated sequences of *OsSNDP3* were fused with the GAL4 DNA-BD of the pGBKT7 vector. To track the bait’s self-transcriptional activity, the constructs were transferred into the haploid Saccharomyces cerevisiae strain AH109 with an empty prey vector and plated on SM media lacking Leu, Trp, and His. The library screening was performed by Panbionet Corp. (http://www.panbionet.com/) using the rice from anther library. Yeast transformants having library plasmid and OsSNDP3 bait were spread on an SD medium lacking leucine, tryptophan, histidine, and adenine (SD-LWHA). Of the 1.13 × 10^7^ colonies, five colonies grew on SD-LWHA plates and the library plasmid was sequenced.

### The RNA Sequencing Analysis

We used mature anthers with trinucleate pollens for RNA-seq analysis. To accomplish this, we created a cDNA library using commercial Illumina library preparation kits (TruSeq Stranded mRNA LT) and three biological replicates were prepared as previously described (Moon et al. 2020). MacroGen Inc. provided the service for the RNA-seq analysis on the Illumina platform. Cutadapt cleaned the raw data, and the cleaned data were mapped to the MSU7 reference genome (RGAP, http://rice.plantbiology.msu.edu/). The raw read counts were then normalized using R packages such as FeatureCounts and DESeq2 (Chandran et al. 2020; Teng et al. 2022). The differentially expressed genes were selected under the following criteria: FPKM in wild type mature anther ≥ 100, log_2_ fold change ≤  − 1; and p-values ≤ 0.05 (Additional file 2: Table S1).

## Supplementary Information

**Additional file 1**: **Fig. S1**. The mutant phenotype of *OsSNDP2*. **Fig. S2**. Mature pollen of *ossndp2 ossndp3-1*.

**Additional file 2**: **Table S1**. Gene lists of downregulated in *ossndp3*. **Table S2**. Classification of gene ontology terms for biological processes associated with 661 genes downregulated in *ossndp3*. **Table S3**. RNA-seq result of three prephenate dehydratases genes that down regulated in *ossndp3*. **Table S4**. RNA-seq result of three pollen-preferential ATPases downregulated in *ossndp3*. **Table S5**. Primer sequences used in this study.

## Data Availability

All supplemental figures and tables are prepared in Additional files.

## Abbreviations

PH: Pleckstrin homology
PIs: Phosphoinositides
PtdIns: Phosphatidylinositol
PtdIns(4,5)P2: Phosphatidylinositol 4,5-bisphosphate
PtdIns4P: Phosphatidylinositol 4-phosphate
PtdIns(3,4,5)P3: Phosphatidylinositol 3,4,5-trisphosphate
PX: Phagocyte oxidase homology
