# Supplementary material for: OsSNDP3 Functions for the Polar Tip Growth in Rice Pollen Together with OsSNDP2, a Paralog of OsSNDP3
Source: Rice (N Y). 2022 Jul 20;15:39. doi: 10.1186/s12284-022-00586-0 (PMC9300783; doi:10.1186/s12284-022-00586-0)
Supplement: Supplementary file 1 — Additional file 1: Fig. S1. The mutant phenotype of OsSNDP2. Fig. S2. Mature pollen of ossndp2 ossndp3-1. [file 12284_2022_586_MOESM1_ESM.docx]

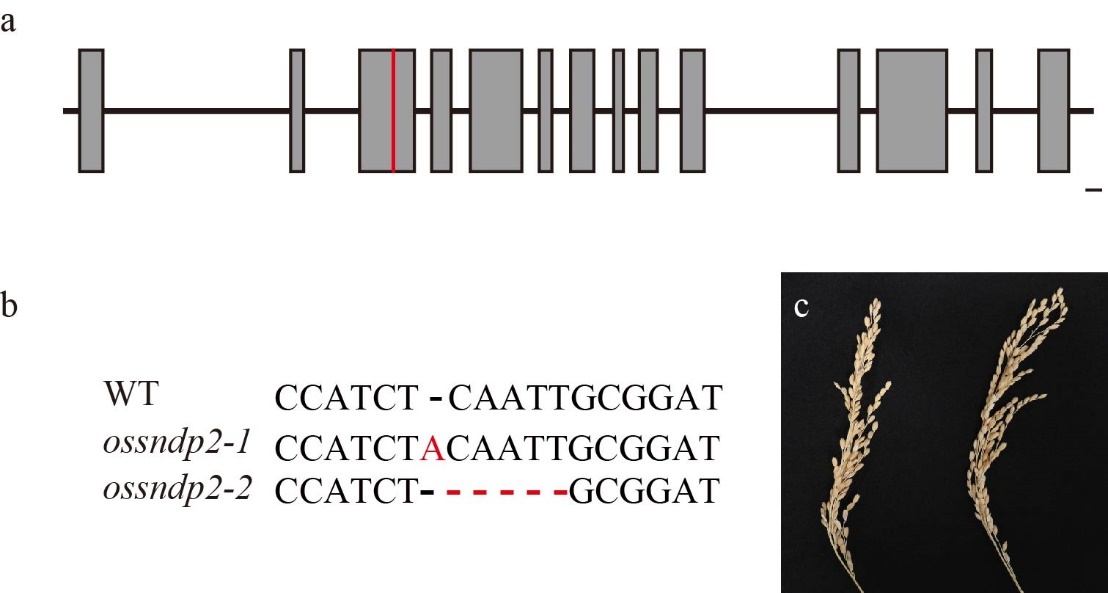


**Figure S1** The mutant phenotype of *OsSNDP2*. **a** Schematic representation of OsSNDP2 including target region for gene-editing. Grey boxes indicate exon; lines, intron; red box, target region of CRISPR/Cas9. **b** Sequencing analysis of the target sites on the genomic regions of *OsSNDP2*. Red character indicates a inserted sequence and red dashes indicate deleted sequences. **c** Panicle from wild type and *ossndp2-1.*


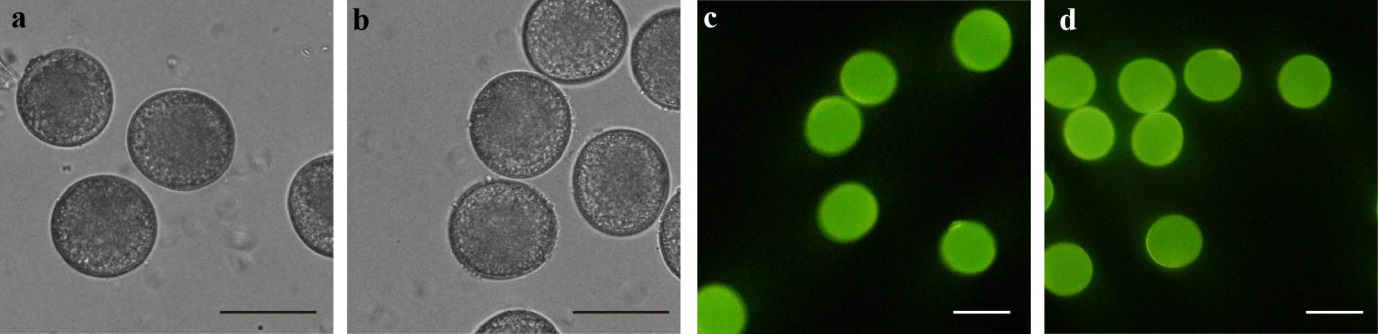


**Figure S2** Mature pollen of *ossndp2 ossndp3.* Pollen in PBS (**a** and **b**) and pollen stained by auramine O (**c** and **d**) from the WT (**a** and **c**) and *ossndp2 ossndp3-1* (**b** and **d**)*.*
